# Role of brain-gut-muscle axis in human health and energy homeostasis

**DOI:** 10.3389/fnut.2022.947033

**Published:** 2022-10-06

**Authors:** Yunju Yin, Qiuping Guo, Xihong Zhou, Yehui Duan, Yuhuan Yang, Saiming Gong, Mengmeng Han, Yating Liu, Zhikang Yang, Qinghua Chen, Fengna Li

**Affiliations:** ^1^College of Animal Science and Technology, Hunan Agricultural University, Changsha, China; ^2^Hunan Provincial Key Laboratory of Animal Nutritional Physiology and Metabolic Process, Key Laboratory of Agro-Ecological Processes in Subtropical Region, Institute of Subtropical Agriculture, Chinese Academy of Sciences, Hunan Provincial Engineering Research Center for Healthy Livestock and Poultry Production, National Engineering Laboratory for Pollution Control and Waste Utilization in Livestock and Poultry Production, Scientific Observing and Experimental Station of Animal Nutrition and Feed Science in South-Central, Ministry of Agriculture, Changsha, China; ^3^College of Advanced Agricultural Sciences, University of Chinese Academy of Sciences, Beijing, China

**Keywords:** energy, glucose, appetite, microbiome, muscle, CNS

## Abstract

The interrelationship between brain, gut and skeletal muscle plays a key role in energy homeostasis of the body, and is becoming a hot topic of research. Intestinal microbial metabolites, such as short-chain fatty acids (SCFAs), bile acids (BAs) and tryptophan metabolites, communicate with the central nervous system (CNS) by binding to their receptors. In fact, there is a cross-talk between the CNS and the gut. The CNS, under the stimulation of pressure, will also affect the stability of the intestinal system, including the local intestinal transport, secretion and permeability of the intestinal system. After the gastrointestinal tract collects information about food absorption, it sends signals to the central system through vagus nerve and other channels to stimulate the secretion of brain-gut peptide and produce feeding behavior, which is also an important part of maintaining energy homeostasis. Skeletal muscle has receptors for SCFAs and BAs. Therefore, intestinal microbiota can participate in skeletal muscle energy metabolism and muscle fiber conversion through their metabolites. Skeletal muscles can also communicate with the gut system during exercise. Under the stimulation of exercise, myokines secreted by skeletal muscle causes the secretion of intestinal hormones, and these hormones can act on the central system and affect food intake. The idea of the brain-gut-muscle axis is gradually being confirmed, and at present it is important for regulating energy homeostasis, which also seems to be relevant to human health. This article focuses on the interaction of intestinal microbiota, central nervous, skeletal muscle energy metabolism, and feeding behavior regulation, which will provide new insight into the diagnostic and treatment strategies for obesity, diabetes, and other metabolic diseases.

## Introduction

The gut microbiome is a complex community, conducive maintaining a dynamic metabolic ecological balance. There are 100 trillion bacteria in adults, of which 80% exist in the intestine, and account for approximately 10 times the number of human cells. The intestinal microbiota contains more than 100 types of bacteria that encode 150 times more genes than the human genome. The human microbiota is composed of more than 1,000 species of microorganisms with over 5,000 strains, including archaea, bacteria, viruses, phages, fungi, protozoa, and nematodes, but mainly bacteria of *Firmicutes* and *Bacteroidetes* (1, 2). Gut microbiome are increasingly recognized as key regulators of host physiology and pathophysiology, and play a vital role in health and disease (3). Changes in the composition of the human gut microbiome are associated with various metabolic diseases, such as obesity, diabetes, and eating disorders (3). Stress-related neuropsychiatric disorders, including depression and anxiety, are also characterized by changes in eating behavior (4). Studies have demonstrated that gut bacteria can influence inflammation, insulin, glucose, and energy metabolism by regulating endocrine signaling pathways along the brain-gut-muscle axis (5). In contrast, a series of CNS diseases caused by stress can also change the composition of intestinal microorganisms through the microbial-gut-brain axis, affect the intestinal epithelial barrier and cause intestinal inflammation (6). Muscle is an endocrine tissue that secretes myokines (e.g., BDNF, FNDC5/irisin, interleukin-6) that control brain functions, including learning, memory, and mood either through the gut or directly through the brain (7, 8). Therefore, in this review, we focus on the intestinal microflora, various signaling pathways affecting the brain-gut-muscle axis, and exploring an effective treatment plan for metabolic diseases such as obesity and diabetes.

## Intestinal microbial metabolites and central nervous system

### Short-chain fatty acids

**FIGURE 1:**
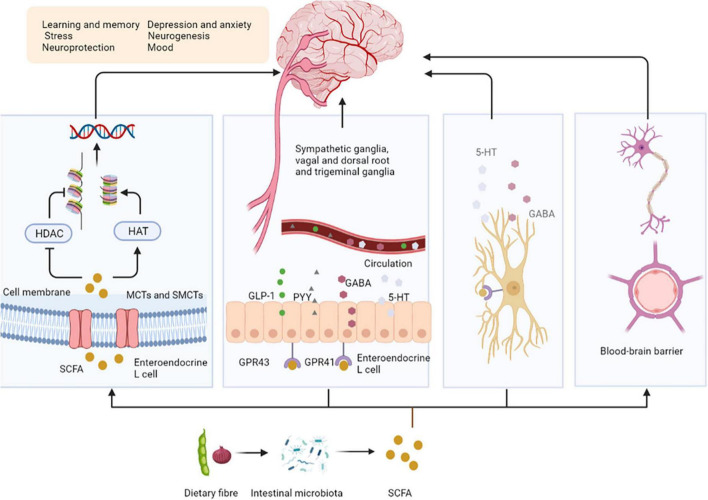
Short-chain fatty acids affect central nervous system activity. SCFAs are absorbed by colonocytes mainly through MCT or SMCTs. By inhibiting the activity of HDACs or increasing the activity of HATs, acetylation can be stimulated to produce chromatin with transcriptional activity and promote gene transcription. SCFAs bind to GPR43, GPR41 on enteroendocrine cells to stimulate their L cells to secrete GLP-1, PYY, GABA, and 5-HT, send signals to the brain. SCFAs can upregulate the expression of tight junction proteins, regulating the growth and development of neurons and synapses. Finally, in the CNS, SCFAs affect the morphology and function of microglia to influence neuro inflammation. SCFAs, short-chain fatty acids; MCTs, H + –dependent monocarboxylate transporters; SMCTs, Na^+^-dependent monocarboxylate transporters; HDACs, histone deacetylases; HATs, histone acetyltransferases; GPR43, free fatty acid receptor 2, GPR41, free fatty acid receptor 3; GLP-1, glucagon-like peptide 1; PYY, peptide YY; GABA, gamma-aminobutyric acid; 5-HT, 5-hydroxytryptamine; BBB, blood-brain barrier.

Dietary fibers enter the colon, and are fermented by microorganisms to produce short-chain fatty acids (SCFAs), which affect the central nervous system (CNS) through various mechanisms (9). After production, SCFAs are absorbed by colonocytes mainly through H^+^-dependent monocarboxylate transporters (MCTs) or Na^+^-dependent monocarboxylate transporters (SMCTs) (10). By inhibiting the activity of histone deacetylases (HDACs) or increasing the activity of histone acetyltransferases (HATs), acetylation can be stimulated to produce chromatin with transcriptional activity and promote gene transcription, thus affecting the brain development and a series of neuropsychiatric diseases such as depression, schizophrenia and Alzheimer’s (11). SCFAs interact with the CNS by binding to free fatty acid receptor 2 (FFAR2 or GPR43), free fatty acid receptor 3 (FFAR3 or GPR41), GPR109a/HCAR2, and GPR164. SCFAs interact with receptors on enteroendocrine cells to stimulate their L cells to secrete glucagon-like peptide 1 (GLP-1), peptide YY (PYY), which indirectly send signals to the brain through the systemic circulation or vagus-nerve pathway (12). SCFAs can cross the blood-brain barrier (BBB) through MCTs located on the endothelial cells, and affecting the BBB integrity by upregulating the expression of tight junction proteins, regulating the growth and development of neurons and synapses, and then affecting the nervous system (13). Finally, in the CNS, SCFAs also affect the morphology and function of microglia, regulate neurotrophic factor levels, increase neurogenesis, promote serotonin biosynthesis, and improve neuronal homeostasis and function to influence neuroinflammation (14) (Figure 1).

### Secondary bile acids

**FIGURE 2:**
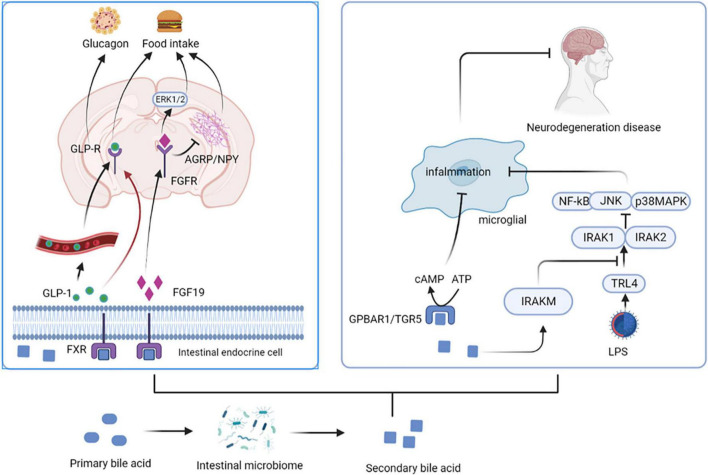
Secondary bile acids affect central nervous system activity. BAs stimulate the release of GLP-1 and FGF19 by activating FXR in the gut. Intestinal GLP-1 activates the brain by activating vagal afferent fibers, influencing feeding behavior and blood sugar levels. FGF19 induces anorexia by binding to FGFR, inducing ERK1/2 signaling and inhibiting AGRP/NPY neuronal activity. UDCA activates GPBAR1 and increases cAMP levels, thereby mediating its anti-inflammatory effect on microglia. In addition, TUDCA induces the transcription of IRAK-M, an inhibitor of the NF-κB pathway, which inhibits the activity of IRAK-1 and IRAK-2 kinases induced by the activation of the LPS-TLR4 pathway. UDCA, ursodeoxycholic acid; TUDCA, tauroursodeoxycholic acid; FGF19, fibroblast growth factor 19; FGFR, fibroblast growth factor receptor; FXR, farnesoid X receptor; ARC, arcuate nucleus; ERK, extracellular signal regular-regulated kinase; AGRP/NPY, gonadotropin-releasing hormone-related peptide/neuropeptide Y; GPBAR1, G protein-coupled bile acid receptor 1; cAMP, cyclic adenosine monophosphate; IRAK, interleukin-1 receptor-associated kinase; NF-κB, nuclear factor kappa-B; LPS, lipopolysaccharide; TLR4, toll-like receptor 4.

Bile acids (BAs) are synthesized from cholesterol in the liver as critical component of bile. The unabsorbed BAs are converted to secondary BAs in the colon by further biotransformation reactions, such as ursodeoxycholic acid (UDCA) and its conjugated derivative tauroursodeoxycholic acid (TUDCA) (13). BAs regulate the permeability of the gut and BBB by disrupting the tight junctions, thereby directly affect brain function (15). They also stimulate the release of GLP-1 and fibroblast growth factor 19 (FGF19) by activating farnesoid X receptor (FXR) in the gut to signal the CNS (16). Intestinal GLP-1 enters the blood and activates the brain by activating vagal afferent fibers, influencing feeding behavior and blood sugar levels (17). FGF19 induces anorexia by binding to receptors expressed in the arcuate nucleus (ARC) of the hypothalamus, inducing extracellular signal regular-regulated kinase (ERK)1/2 signaling and inhibiting gonadotropin-releasing hormone-related peptide/neuropeptide Y (AGRP/NPY) neuronal activity (18). BAs can also affect immune responses; for example, UDCA activates G protein-coupled bile acid receptor 1 (GPBAR1) expressed on microglia and increases cyclic adenosine monophosphate (cAMP) levels, thereby mediating its anti-inflammatory effect on microglia (13). In addition, TUDCA induces the transcription of interleukin-1 receptor-associated kinase M (IRAK-M), an inhibitor of the nuclear factor kappa-B (NF-κB) pathway, which inhibits the activity of IRAK-1 and IRAK-2 kinases induced by the activation of the lipopolysaccharide (LPS)-toll-like receptor 4 (TLR4) pathway. These and other pro-inflammatory pathways are prerequisites for activating downstream targets such as NF-κB, c-Jun N-terminal kinase (JNK), and p38RK. Therefore, TUDCA inhibits the pro-inflammatory pathway by promoting the expression of NF-κB inhibitors (such as IRAK-M) (19) (Figure 2).

### Tryptophan metabolites

**FIGURE 3:**
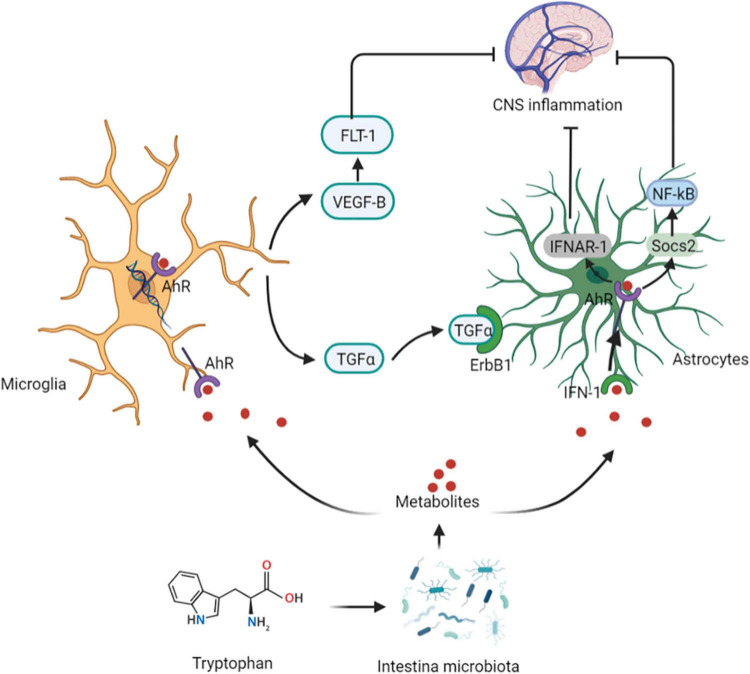
Tryptophan affects central nervous system activity. The protective effect of the AhR ligand on CNS autoimmunity is through the IFN-I-AhR-Socs2-NF-κB signaling pathway. Microglia can send signals to astrocytes to mediate the response to the inflammatory in CNS. The microbial metabolites of tryptophan regulate the microglial activation, produce TGFα and VEGF-B, regulate CNS-related diseases, and the transcription in astrocytes through AhR. Microglia derived-TGFα can bind to the ErbB1 in astrocytes to show exert neuroprotective effects. In contrast, VEGF-B production triggers FLT-1 signaling in astrocytes. AhR, aryl hydrocarbon receptor; IFN-I, Type I interferon; Socs2, suppressors of cytokine signaling 2; IFNAR-1, AhR-mediated interferon-α receptor-1; TGFα, transforming growth factor-alpha; VEGF-B, vascular endothelial growth factor B; ErbB1, epidermal growth receptor; FLT-1, endothelial growth factor receptor 1.

Intestinal microbiota can metabolize Tryptophan in dietary protein by intestinal microbiota to produce 5-HT, kynurenine, tryptophan, and indole compounds (20). Which affect CNS inflammation and participate in neuropsychiatric diseases through the aryl hydrocarbon receptor (AhR) (21). It has been suggested that 5-HT, can be used as a ligand of AhR (22). Type I interferon (IFN-I) combines with microbial tryptophan metabolites in astrocytes to activate AhR (23). Activation of AhR subsequently inhibits NF-κB activation by inducing the expression of suppressors of cytokine signaling 2 (Socs2), thereby inhibiting the inflammatory response. In addition, AhR-mediated interferon-α receptor-1 (IFNAR-1) also shows a similar effects by inhibiting inflammation and neurodegeneration (24). Ahr gene knockout in astrocytes can lead to deterioration of CNS autoimmunity. Therefore, the protective effect of the AhR ligand on CNS autoimmunity is through the IFN-I-AhR-Socs2-NF-κB signaling pathway (25). In addition to astrocytes, microglia are immune cells of the CNS that can express AhR (26). Microglia can send signals to astrocytes to mediate the response to the inflammatory in CNS (27). The microbial metabolites of tryptophan regulate the microglial activation, produce transforming growth factor-alpha (TGFα) and vascular endothelial growth factor B (VEGF-B), regulate CNS-related diseases, and the transcription in astrocytes through AhR (28). In-depth studies have shown that microglia derived-TGFα can bind to the epidermal growth receptor (ErbB1) in astrocytes to play a neuroprotective role and promote their beneficial activities (29). In contrast, VEGF-B production triggers vascular endothelial growth factor receptor 1 (FLT-1) signaling in astrocytes, which intensifies its pathogenic activity and worsens the experimental allergic encephalomyelitis (EAE) development. Additionally, VEGF-B and TGFα are also involved in the formation of multiple sclerosis (MS) lesions in CD14 + cells, and participate in the microglial control of astrocytes (28) (Figure 3).

## Effects of central nervous system on the intestinal tract

The ability of the intestinal epithelium to act as a barrier between the external and tightly regulated internal environments is critical for human health. The brain can influence the structure and function of the gut microbiome through the autonomic nervous system (ANS), by regulating local intestinal movement, transport and secretion, and permeability (6). The brain-gut axis is a two-way communication network between gut and brain. Communication occurs through three different pathways: the nervous system pathway (30), the endocrine pathway, and the immune pathway (31).

### Nervous system pathway

Stress directly stimulates the chromaffin cells in the medulla through sympathetic nerves to release norepinephrine (NA) and activates neurons in the myenteric plexus α2-adrenergic receptors (ARs), resulting in α2-AR mediated inhibition of intestinal motility through presynaptic and postsynaptic signaling mechanisms (32). Additionally, submucosal neurons α2-ARs signaling in the submucosal neurons leads to decreased mucosal electrolyte secretion (33). However, the activation of β3-ARs leads to somatostatin release from the colon and inhibits cholinergic-mediated colonic contraction. This may improve the injury and improve the intramural blood flow in the chemical-induced inflammation model, further promoting mucosal healing (34). Therefore, indirect effects contribute to β3-AR-mediated signaling and confers inflammatory protection (35). Similarly, the regulation of inflammation by α2-ARs may be due to the direct pro-inflammatory effect of α-2-Ars activation on immune cells, or the indirect effect of presynaptic inhibition of α-2-AR-mediated norepinephrine release (36). Changes in the transepithelial fluid and ion transport are thought to play a role in intestinal defense (37). In the mucosa of the small intestine of many mammals, NA or adrenaline increases the active absorption of Na^+^ ions, which further increases the absorption of water from the intestinal cavity. These effects are usually caused by α-ARs located in the intestine and their projection toward the epithelial cells (38). However, these catecholamines can act on α- or β- Ars in the colon, which causes the secretion of potassium, bicarbonate, or chloride ions (39).

### Endocrine pathway

Psychological and physiological stress activates the hypothalamic-pituitary-adrenal (HPA) axis, leading to the release of corticotropin-releasing hormone, which is the main regulator of the HPA axis. Corticotropin releasing factor (CRF) is secreted by small cell neurons located in the paraventricular nucleus of the hypothalamus. CRF promotes the synthesis and release of the anterior pituitary hormone (ACTH), which acts on the adrenal gland to promote the synthesis of glucocorticoids (GCs) (40). The GCs influence lymphocytes in Peyer’s patches to undergo apoptosis, accelerates the pro-inflammatory responses. reduces the production of plasma cells and IgA antibodies, which destroys intestinal immunity. Additionally, GCs inhabit tight junction proteins (TJP), resulting in increased intestinal permeability and bacterial transfer to the lamina propria, which further promotes the inflammatory process (41). According to a study, GC prevented the release of TNF by inhibiting the transcription of myosin light chain kinase-α. GCs-induced increase in TJ permeability of the intestinal epithelium enhances the function of epithelial barrier (42). On the other hand, it has been found that restraint stress in mice increases the expression of heat shock protein-70 (HSP-70) in colonic epithelial cells through the interaction between LPS and TLR4, leading to the expression of type 1 closed band (zona occludens; ZO-1) protein. A glucocorticoid receptor antagonist mifepristone can inhibit the pressure-induced changes in colonic LPS permeability and the expression of HSP-70 and ZO-1 (43, 44). Mast cells communicate with the intestinal tract, autonomic nerves and CNS through mast cell mediators and neuropeptides (45). Under pressure stimulation, mast cells secrete CRH that binds to the expression of CRF receptor 1 in goblet cells, promoting their apoptosis and mucus depletion (46), and promotes intestinal inflammation (45).

### Immune pathway

The immune pathway is also an important component of the two-way communication between the brain and intestine, which affects intestinal barrier function by regulating the secretion of cytokines. Acute and repeated stress can affect the intestinal secretion of IgA, intestinal homeostasis, and lead to inflammation (47). Changes in the IgA levels in the intestine may also lead to changes in symbionts and may lead to ecological imbalance. Secretory immunoglobulins, especially secretory IgA (S-IgA), are transported to the lumen through epithelial cells and play an important role in mucosal protection and localization of symbiotic microbial community on the intestinal surface (48). Adrenergic nerves are located in the small and large intestine near IgA positive B lymphocytes and crypt epithelial cells containing polymeric immunoglobulin receptor (pIgR). NE rapidly stimulates the release of S-IgA in the mucosal layer of any intestinal segment owing to the increased number of epithelial pIgRs (49). Restraint stress in rats can increase the IgA levels in the lamina propria of small intestine, which is inhibited by 6-hydroxydopamine chemical sympathectomy (50). This inhibition reduced the number of IgA-positive lamina propria cells in weaned rats (51). In contrast, glucocorticoid utilization is associated with increased or decreased in intestinal IgA levels, depending on the species (50).

## Brain-gut peptides

Neurons in the gastrointestinal tract can stimulate these tissues to produce a range of brain-gut peptides based on the composition of ingested food. By acting as appetite regulator and dietary intermediates between the gastrointestinal tract and brain, brain-gut peptide such as PYY, ghrelin, GLP-1, provide powerful targets for treating of obesity. Overall energy intake is controlled by a series of brain-gut peptides that activate the energy regulation center of the CNS by activating endocrine and nervous signals (52).

### Glucagon-like peptide 1

**FIGURE 4:**
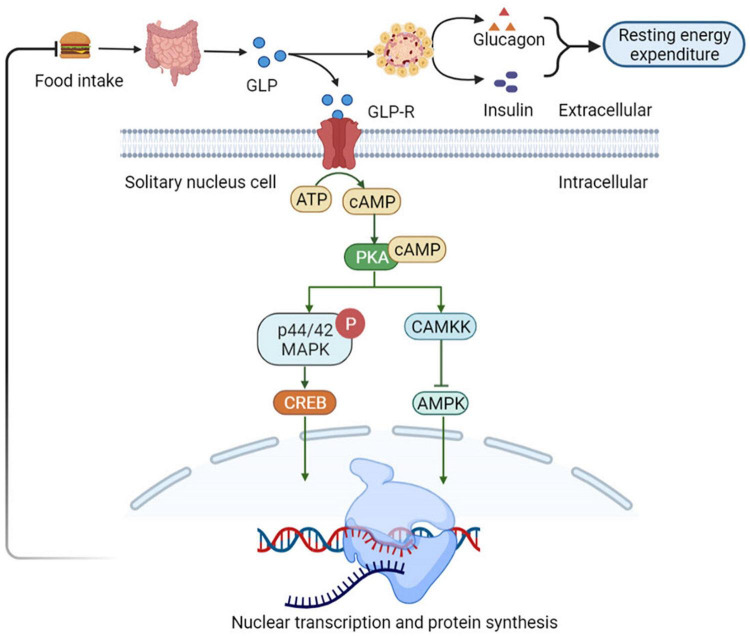
Glucagon-like peptide 1 (GLP-1) regulates appetite and energy metabolism. GLP-1R-expressing neurons to initiate a cAMP-dependent increase in PKA activity, drives simultaneous phosphorylation and activation of p44/42 MAPK and inhibits CaMKK. It catalyzes the phosphorylation of Thr172 residue in the AMPKα subunit, resulting in CAMKK activity inhibition and reduced AMPK activation, which promote an elevated in CREB-mediated nuclear transcription and protein synthesis. In this way the NTS GLP-1R-expressing neurons can integrate various anorectic signals involved in meal-to-meal food intake control. NTS, nucleus of the solitary tract; PKA, protein kinase A; MAPK, mitogen-activated protein kinase; CaMKK, Ca^2+^/calmodulin-dependent protein kinase kinase; AMPKα, adenosine monophosphate protein kinase α; CREB, cAMP response element-binding.

Nutrients intake can stimulate the GLP-1 secretion by intestinal L cells, which activates GLP-1 receptors (GLP-1R) present in the gastrointestinal tract hepatic portal vein. The activation of these receptors triggers vagal reflex, increases efferent pancreatic vagus nerve activity, and stimulates insulin release (53). Insulin, in turn, increases the resting energy expenditure by inducing sympathetic activation and glucose utilization, thereby increasing the thermic effect of glucose (54). Simultaneously, GLP-1 inhibits glucagon-induced increases in resting energy expenditure, reduces gastric emptying, and nutrient absorption. Therefore, it plays a satiating role by decreasing the insulin response and thermic effect of food (55). Consequently, it sends signals to the tuberous ganglion to activate the nucleus tractus solitarii (NTS) neurons in the brain acts on the endemic NTS GLP-1R-expressing neurons to initiate a cAMP-dependent increase in protein kinase A (PKA) activity (56), drives simultaneous phosphorylation and activation of p44/42 mitogen-activated protein kinase (MAPK) and inhibits calcium/calmodulin-dependent protein kinase kinase (CaMKK) (57), inhibition of CAMKK activity resulting in reduced AMPK activation (57). Increased PKA and p44/42 MAPK activities together with a decrease in AMPK activity promote an elevated in cAMP response element-binding (CREB)-mediated nuclear transcription and protein synthesis. In thus way the NTS GLP-1R-expressing neurons can integrate various anorectic signals involved in meal-to-meal food intake control (58) (Figure 4).

### Peptide YY

Peptide YY, also known as peptide tyrosine, which also includes NPY, and pancreatic polypeptide (PP) secreted by the L cells of the colon and small intestine in response to local nutritional stimulation. PYY_3–36_ is the main form present in the intestinal mucosal endocrine cells and circulation, with the highest affinity for Y2 receptors (59). Batterham et al. (60)showed that the peripheral injection of PYY_3–36_ inhibited food intake and weight gain in rats. It also inhibited food intake in mice but not without Y2 receptors, suggesting that the anorexia effect requires a Y2 receptor (60). Peripheral administration of PYY_3–36_ increased c-FOS immunoreactivity in the ARC and decreased NPY mRNA expression in the hypothalamus. Furthermore, when dietary intake, high levels of PYY freely cross the BBB and inhibit the Y2 receptor, which is heavily expressed within the ARC of the hypothalamus. Thus, PYY also inhibits the electrical activity of NPY nerve endings, thereby activating the adjacent proopiomelanocortin (POMC) neurons and regulating food intake and energy homeostasis. However, studies have shown that the action potential firing activity of POMC neurons of the ARC is inhibited through postsynaptic Y2 receptors (61). Although the peripheral administration of PYY reduced food intake and weight gain in mice, this effect disappeared with Y2 receptor knockout (62). Since PYY and Y2 receptors are expressed by myenteric plexus neurons and vagus nerve, respectively, the vagal brainstem mediated pathways may also be involved (59). Research evidence suggests that higher levels of PYY_3–36_, but not PYY_1–36_, reduced food consumption and hunger with increased satiety levels of satiety (63). PYY_3–36_ acts as a signaling factor for anorexia, satiety, and acts locally to delay gastrointestinal transport and emptying (64).

### Ghrelin

**FIGURE 5:**
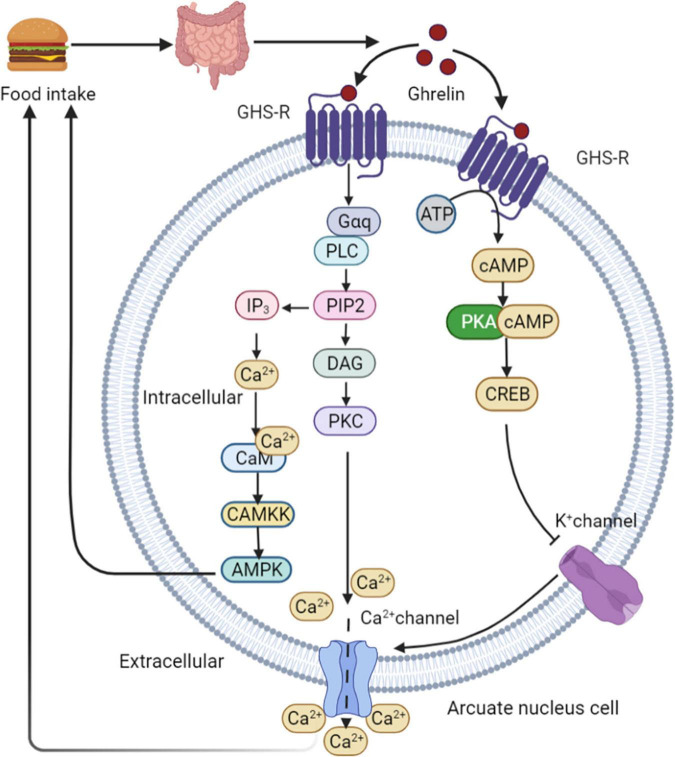
Ghrelin mediated appetite regulation. PKA is activated by ghrelin in a cAMP-dependent manner. PKA phosphorylates CREB, thereby inhibiting potassium channels, depolarizing cells, and opening N-type calcium channels. Ca^2+^ are then released from intracellular storage, indicating ghrelin-mediated appetite regulation. High calcium levels induce a second cascade of reactions involving PLC, which triggers the hydrolysis of PIP2 in the cell membrane. This process leads to increased intracellular calcium levels in two ways. First, PIP2 activates DAG for PKC activation through tyrosine phosphorylation, resulting in the opening of L-type calcium channels. Second, IP3 activated by PIP2 induces the rapid release of calcium from the endoplasmic reticulum. Increased intracellular Ca^2+^ levels interact with CAM to activate CAMKK, an upstream kinase that activates AMPK and stimulates appetite. GHS-R, growth hormone secretagogue receptor; PLC, phospholipase C; PIP2, phosphatidylinositol-4,5-diphosphate; DAG, diacylglycerol; PKC, protein kinase C; IP3, inositol triphosphate; CAM, calmodulin.

Ghrelin is produced by endocrine cells in the stomach and duodenum. Ghrelin release depends on the regulation of food intake. The highest ghrelin are presented in the empty stomach and decrease rapidly after eating (65). The binding of ghrelin to growth hormone secretagogue receptor 1A (GHS-R1A) leads to the activation of two calcium signaling cascades, triggering various cellular responses. This process initiates metabolic processes such as appetite changes and regulation of glucose homeostasis. In the first signaling pathway, PKA is activated in a cAMP-dependent manner. PKA phosphorylates CREB, thereby inhibiting potassium channels, depolarizing cells, and opening N-type calcium channels. Ca^2+^ ions are then released from intracellular storage, indicating ghrelin-mediated appetite regulation (66). High calcium levels induce a second cascade of reactions involving phospholipase C (PLC), which triggers the hydrolysis of phosphatidylinositol-4,5-diphosphate (PIP2) in the cell membrane. This process leads to increased intracellular calcium levels in two ways. First, PIP2 activates diacylglycerol (DAG) for protein kinase C (PKC) activation through tyrosine phosphorylation, resulting in the opening of L-type calcium channels. Second, inositol triphosphate (IP3) activated by PIP2 induces the rapid release of calcium from the endoplasmic reticulum (67). Increased intracellular Ca^2+^ levels interact with calmodulin (CAM) to activate CAMKK, an upstream kinase that activates AMPK and stimulates appetite (68). Downstream intracellular action of ghrelin following AMPK activation involves phosphorylation of acetyl-CoA carboxylase (ACC), which results in malonyl-CoA inhibition and carnitine palmityl transferase 1 (CPT1) activation (69), lead to increased food consumption (69, 70). After binding to its receptor, ghrelin activates the AMPK-CPT1-UCP2 axis, which is important for mitochondrial biogenesis in NPY/AgRP neurons. Electrical activation of NPY/AgRP neurons and ghrelin triggers the POMC synaptic plasticity. Taken together, these results suggest that ghrelin indirectly stimulates UCP2 by activating AMPK. This cascade stimulates NPY/AgRP neurons and initiate the appetite process eventually (71). In healthy individuals, ghrelin administration reduces GSIS and glucose tolerance (72). Ablation of the ghrelin gene increases GSIS and improves insulin sensitivity in HFD fed mice and leptin-deficient obese (OB/OB) mice (73) (Figure 5).

## Gut–Muscle axis

### Energy regulation

Intestinal microbiota affects the development and function of the CNS, and regulate appetite and energy homeostasis. Additionally, they regulate skeletal muscle metabolism through metabolites, such as SCFAs and BAs, thereby regulating animal energy homeostasis. SCFAs can induce the AMPK phosphorylation in myotubes and skeletal muscles, owing to their ability to increase AMP concentration and the AMP/ATP (74). Phosphorylated AMPK activates its downstream targets, including p38MAPK and peroxisome proliferator-activated receptor γ coactivator-1-α (PGC-1 α) (75). The PGC-1-α promotes mitochondrial biosynthesis, enhances its function, and the oxidative metabolism of fatty acids (76). AMPK elevated the expression of the glucose transporter (GLUT4) phosphorylating of HDAC5, thereby inducing the release of HDAC5 from the nucleus of human primary myotubes (77). Glucose diffuses into the muscle cells through GLUT4, leading to enhanced muscle glycogen storage (78). In addition to the above pathways, SCFAs can bind to GPR41 and GPR43 receptors to stimulate colonic L cells and pancreatic tissue to release GLP-1 and insulin, respectively (79). GLP-1 signaling through GLP-1R promotes PI3K activation and increases GLUT4 protein levels in the cytoplasm, resulting in increased glucose absorption by cells. GLP-1 also stimulates glucose uptake independent of PKB phosphorylation and promotes its storage independent of GSK3 phosphorylation (80). Therefore, GLP-1 can increase the aggregation of skeletal muscle microvessels, glucose uptake, metabolism and glycogen synthesis (81). Insulin increases the rate of glycolysis by increasing glucose transport and the hexokinase, fructokinase 6-phosphate (82). The increased AMPK activity inhibited the expression of lipogenesis genes ACC, SREBP-1c and FAS. It increases the expression of CPT1, U, LPL, and HSL genes, resulting in enhanced lipid catabolism and reduced muscle fat accumulation (83). Moreover, BAs produced by intestinal microbiota can be used as signaling molecules to regulate G protein-coupled bile acid receptor 1 (TGR5) in muscles, promote the activation of thyroid hormone, and improve the energy consumption of human skeletal muscle cells (84). Additionally, BAs prevent muscles fat deposition by activating the nuclear FXR (85). In conclusion, intestinal microorganisms can produce metabolites that regulate muscle lipid metabolism and glucose homeostasis, which also provide new insights into the treatment of type 2 diabetes and obesity.

### Regulatory muscle fiber transformation

**FIGURE 6:**
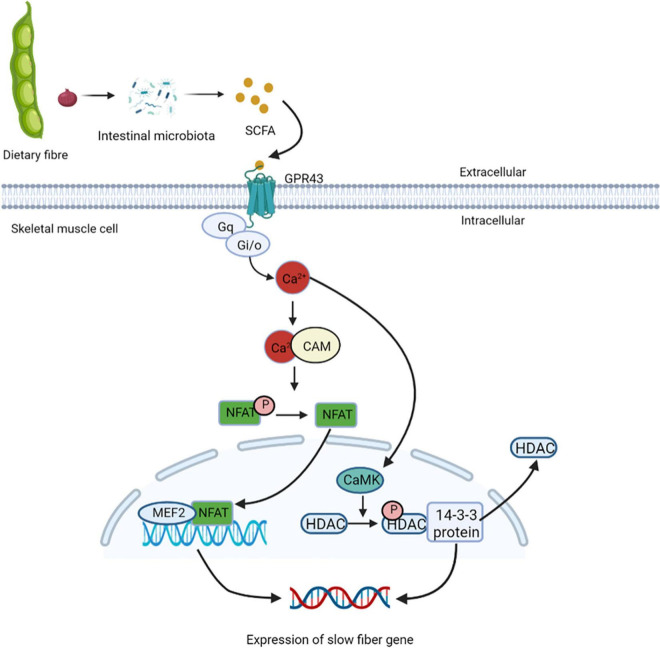
Short-chain fatty acids regulate the expression of slow muscle fiber genes. GPR43 is activated by treatment with acetic acid, and coupled to Gq and Gi/o protein families, it can enhance the production of IP3 and intracellular Ca^2+^, and inhibit the accumulation cAMP. Elevated intracellular Ca^2+^ levels activate further downstream the Ca^2+^/CAM-dependent phosphatase calcineurin pathway. Calcineurin is activated after binding to Ca^2+^/CAM, which gets NFAT enter the nucleus and binds to nucleotide recognition sequences and activating slow muscle fiber genes. In the nucleus of skeletal muscles, NFAT and MEF2 interact with target genes, control the transcription of genes encoding slow-fiber program proteins. The increased Ca^2+^ levels inside the cytoplasm promote it enter into the nucleus and activates intracellular CaM kinase, which phosphorylates HDACs in the nucleus, binds phosphorylated HDACs to the 14-3-3 protein, and moved them. This relieves the inhibition of MEF2 transcriptional activity and activates the expression of slow-fiber gene. NFAT, nuclear factor of activated T cells; MEF2, myocyte enhancer factor 2; CaMK, calmodulin-dependent kinase.

Studies have found that cross innervation, some disease states, or lack of gravity and physical activity reduce motor nerve activity, leading to the transformation of fibers from slow to fast (86). In skeletal muscles, the increased intracellular calcium influx caused by exercise stimulation plays a key role in the contractile activity-dependent expression and fiber-type specific gene expression (87). GPR43 is expressed in skeletal muscle cells and is activated acetic acid (88). Studies have also shown the presence of nuclear factor of activated T cells (NFAT) recognition sites upstream of GPR43 gene, and NFAT controls the expression of GPR43 and other muscular oxidative fiber genes (89). GPR43 is coupled to Gq and Gi/o protein families, it can enhance the production of signaling molecules, such as IP 3 and intracellular Ca^2+^ ions, and inhibit the accumulation cAMP (90). Elevated intracellular Ca^2+^ levels activates further downstream signaling pathways, including the Ca^2+^/CAM-dependent phosphatase calcineurin pathway (91). Calcineurin is activated after binding to Ca^2+^/CAM. Once dephosphorylated by calcineurin, NFAT exposes nuclear localization signals on their surface, and enters the nucleus (92). NFAT bind to nucleotide recognition sequences and resulting in nuclear NFAT mediated activation of slow muscle fiber genes (93). In the nucleus of skeletal muscles, NFAT with other transcription regulators such as myocyte enhancer factor 2 (MEF2), interact with target genes. The binding sites of MEF2 are clustered gather in the promoter/enhancer region to control the transcription of genes encoding slow-fiber program proteins (94). The increased Ca^2+^ levels inside the cytoplasm promote it enter into the nucleus through the nuclear pore complex and activates intracellular CaM kinase. Activated nuclear calmodulin-dependent kinase (CaMK) phosphorylates HDACs in the nucleus, binds phosphorylated HDACs to the 14-3-3 protein, and moved them. This relieves the inhibition of MEF2 transcriptional activity and activates the expression of slow-fiber genes (95) (Figure 6). Eventually, discovery of calcineurin regulatory pathways controlling skeletal muscle fibers may serve as a new strategy to improve human health.

### Effects of key nutrients on the gut-muscle axis

The dietary fibers are fermented by microorganisms in the colon to produce SCFAs, such as acetic, butyric and propionic acids. In particular, the skeletal muscle cells use acetate to produce ATP, whereas the metabolic fate of butyrate and propionate is mainly related to gluconeogenesis and cholesterol synthesis (96). Carbohydrates stimulate the intestinal GLP-1, which plays a role in insulin secretion and improve the glycogen reserves in skeletal muscles (97) (Table 1).

**TABLE 1:** Key nutrients regulate muscle fiber transformation.

Nutrients	Dose	Subject	Effects	References
Butyrate	5%	Mice	Induction of type I fiber differentiation in skeletal muscles, a 200% increase in fatty acid oxidation in the gastrocnemius muscle increased transcription and expression of PGC-1α in both mRNA and protein, respectively.	Gao et al. (98)
Butyrate	80 mg	Mice	Decreased concentrations of skeletal muscle triglycerides and cholesterol enhance the expression of adiponectin receptors (adipoR1/2) and AMP kinase (AMPK), while reducing the expression of histone deacetylase 1 (HDAC1), significantly increasing the muscle content of ADP and AMP.	Hong et al. (99)
Butyrate	5%	Mice	Reduced muscle fiber cross-sectional area and prevented intramuscular fat buildup in older mice, reduced fat mass and improved glucose metabolism in 26-month-old mice, increasing markers of skeletal muscle mitochondrial biogenesis.	Walsh et al. (100)
Acetic acid	10 mL/kg body mass	Mice	The gene expression of AMPK and PPARδ, LKB1, pAMPK, and PGC-1α in the mouse soleus increased, resulting in increased expression of MHCI, MHCIIa and decreased expression of MHCIIb.	Pan et al. (101)
Acetic acid	0.5 Mm	L6 myoblasts	Enhances glucose uptake and fatty acid metabolism by activating AMPK, increases the expression of GLUT4 myoglobin, MEF2A-related genes and proteins.	Maruta et al. (74)
Propionate	300 μM	C2C12 Myotubes	Basal glucose uptake in the muscle tubes.	Han et al. (102)

## Brain-gut-muscle-axis

### Brain-gut-muscle-axis in exercise

**FIGURE 7:**
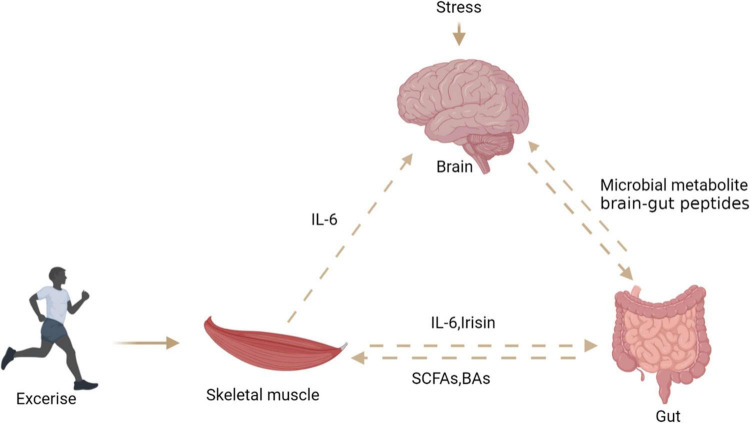
The relationship between brain, gut, and skeletal muscle. Exercise can stimulate skeletal muscle to secrete interleukin-6 and irisin, which act on intestinal endocrine cells to secrete PYY, stimulate the central system and affect appetite. Stress stimulates the central system to affect the intestinal barrier through the HPA axis. Intestinal microbial metabolites can directly or indirectly affect the central system activity and skeletal muscle energy metabolism.

Skeletal muscle is considered as an endocrine organ due to proteins expressed and secreted by myotubes known as myokines. Exercise seems to connect muscles, intestines, and muscles (Figure 7). Skeletal muscle may produce hundreds of proteins, among which IL-6 and IL-15 has attracted great attention (103). Ellingsgaard et al. (104) found that the systemic IL-6 concentration of mice increased after exercise, and this increase was mediated by skeletal muscle-derived IL-6. The increase of IL-6 concentration stimulates intestinal L cells to secrete GLP-1, which acts on the CNS through circulation, and then affects appetite, IL-6 can also act directly on CNS to regulate appetite. Irisin, a novel myokine, is sharply upregulated by exercise (7). Studies have shown that central irisin injection can increase food intake and ghrelin levels in rats. It is speculated that muscle-derived irisin can stimulate gastrointestinal tissue to secrete ghrelin and affect appetite in the CNS (105). A recent study found that exercise stimulates the production of N-lactoyl-phenylalanine (Lac-Phe), a blood-borne signaling metabolite that suppresses feeding and obesity (106). Several studies reported that participants’ plasma PYY and PP concentrations increased, which inhibited hunger in a short time (107–109). Therefore, the phenomenon of “exercise-induced anorexia” may be related to the increased levels of PYY, GLP-1 and PP observed during exercise (107). Most studies have revealed that myokines secreted after exercise can stimulate gastrointestinal hormone secretion and affect appetite in the central system. However, the mechanism of how muscle cytokines act on intestinal endocrine cells is not clear, and further research is needed to clarify the mechanism of their cross-talk.

### Brain-gut-muscle-axis in pathological condition

Diseases of the CNS lead to an imbalance in the intestinal microbiota and a decrease in the production of SCFAs, leading to an increase in intestinal permeability (110), this may help facilitate the transfer of microbial byproducts into the cycle. Microbial byproducts include endotoxins, such as LPS; They can induce chronic inflammation throughout the body as well as insulin resistance, a form of metabolic resistance that ultimately leads to sarcopenia (111, 112). In addition, SCFAs bind to receptors 2 and 3 (FFAR 2/3) on skeletal muscle through circulation, and release insulin-like growth factor (IGF-1) through mechanisms that promote glucose uptake and metabolism. This protein binds to insulin receptor substrate 1 (IRS1) and activates the phosphorylated inositol 3-kinase (PI3K) –Akt mammalian target of rapamycin (mTOR) pathway, stimulating protein synthesis in skeletal muscle tissue and blocking protein hydrolysis (113). In the pathological conditions, the production of SCFAs is reduced, and the level of IGF-1 is low. IGF-1 impairs protein synthesis by inhibiting PI3K-Akt-mTOR pathway, resulting in sarcopenia (114). Low concentrations of SCFAs in the gut have been shown to be associated with increased subclinical chronic inflammation, which then leads to sarcopenia (115).

## Summary

Gut microbiome collaborate with their animal hosts to regulate immune, metabolic, nervous system, and muscle development and function through dynamic bidirectional communication along the brain-gut-muscle axis. Brain-gut peptide and myokines are mediators of brain-gut-muscle axis interaction. Further studies of the brain-gut axis will shed light on the pathogenesis of neurodegenerative diseases (such as Parkinson’s disease) and metabolic diseases. The brain-gut-muscle axis is the basis for studying sarcopenia and finally evaluating the potential of microbiome targeted therapies.

## Author contributions

QG, XZ, YD, YYa, SG, MH, YL, ZY, QC, and FL are responsible for collecting data and wrote the manuscripts. All authors contributed to the article and approved the submitted version.
